# Supplementary material for: Effects of a natural ingredients-based intervention targeting the hallmarks of aging on epigenetic clocks, physical function, and body composition: a single-arm clinical trial
Source: Aging (Albany NY). 2025 Mar 14;17(3):699–725. doi: 10.18632/aging.206221 (PMC11984428; doi:10.18632/aging.206221)
Supplement: Supplementary Figures [file aging-17-206221-s001.pdf]

SUPPLEMENTARY FIGURES

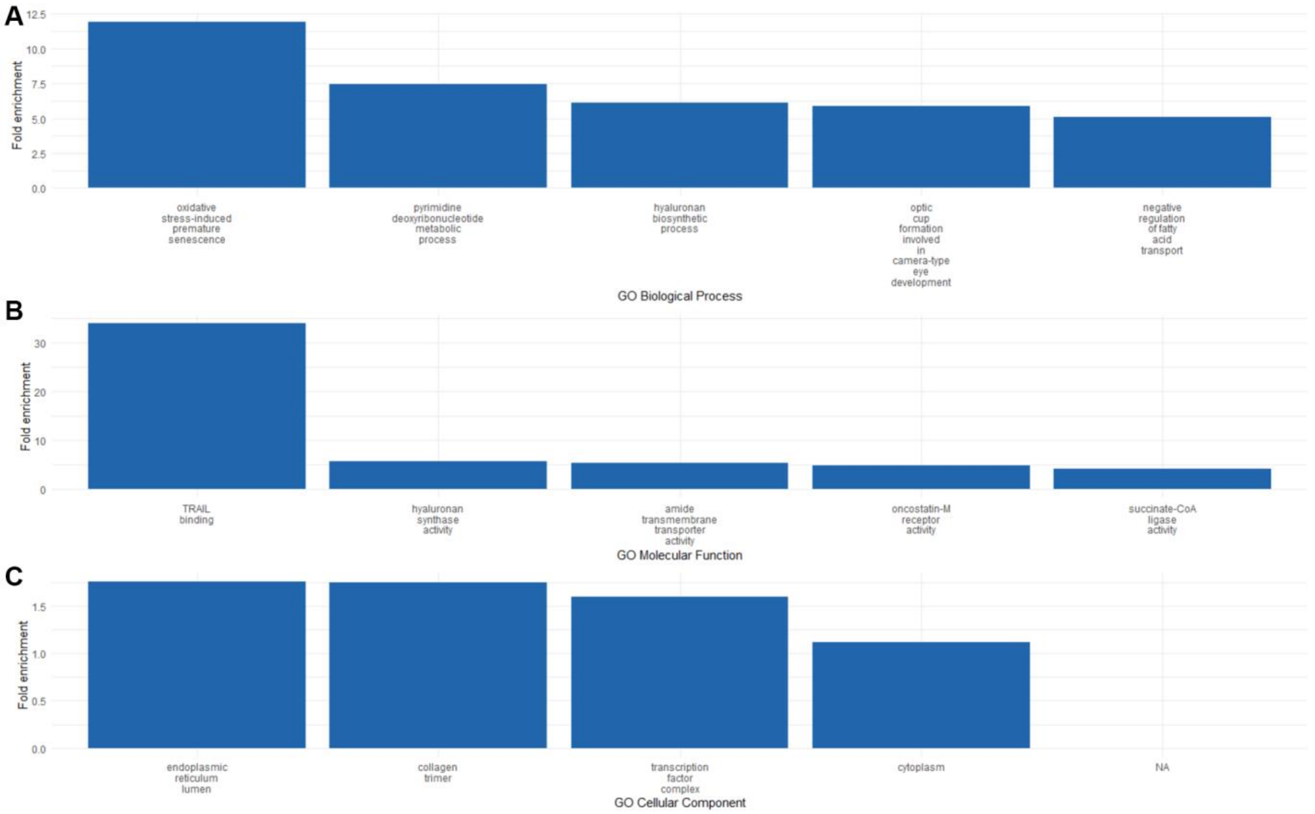

Supplementary Figure 1. Gene Ontology (GO) terms enriched for the hypermethylated CpG sites after supplementation. The Top 5 most enriched GO terms for Biological Processes (**A**), Molecular Function (**B**), and Cellular Component (**C**) were included. A nominal *p*-value <0.001 was used to assign significance.

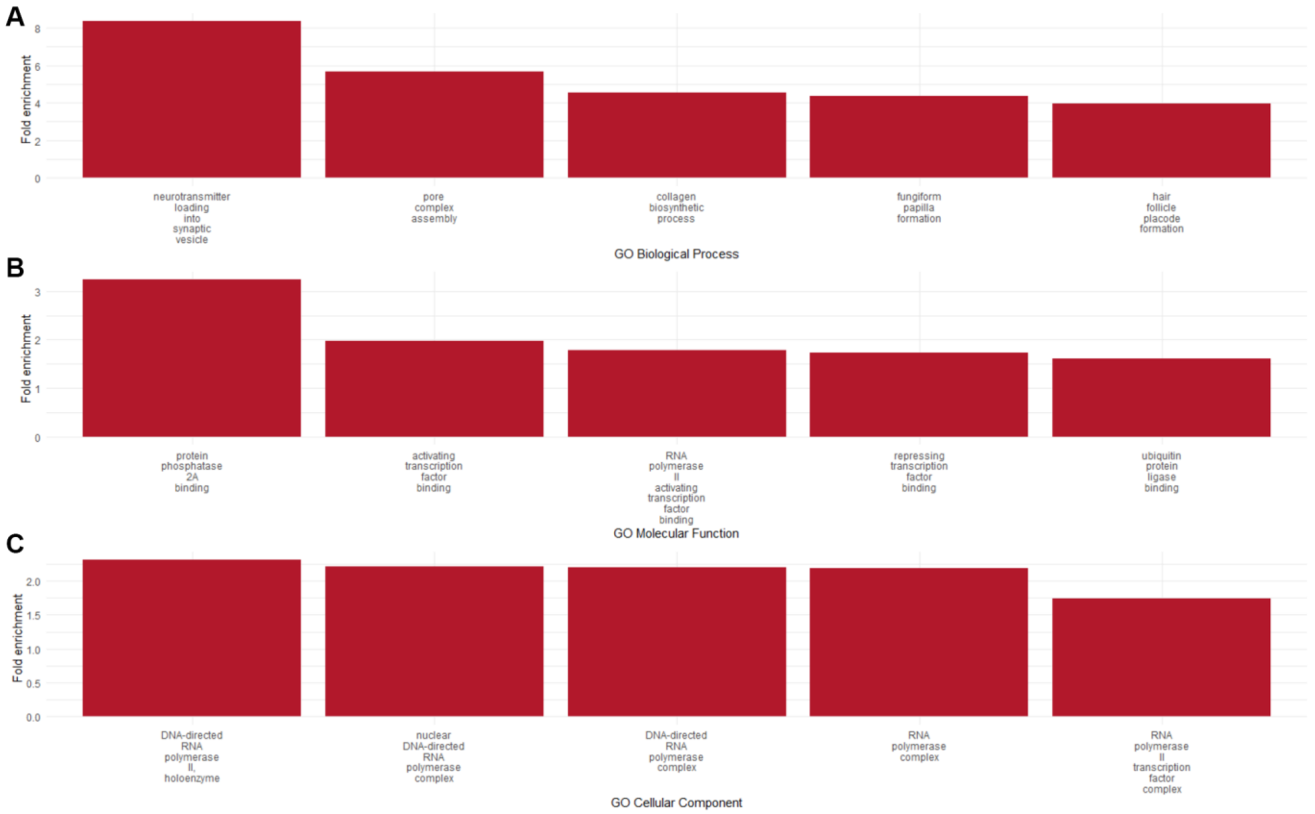

Supplementary Figure 2. Gene Ontology (GO) terms enriched for the hypomethylated CpG sites after supplementation. The Top 5 most enriched GO terms for Biological Processes (**A**), Molecular Function (**B**), and Cellular Components (**C**) were included. A nominal *p*-value <0.001 was used to assign significance.
