# Supplementary material for: Effects of a natural ingredients-based intervention targeting the hallmarks of aging on epigenetic clocks, physical function, and body composition: a single-arm clinical trial
Source: Aging (Albany NY). 2025 Mar 14;17(3):699–725. doi: 10.18632/aging.206221 (PMC11984428; doi:10.18632/aging.206221)
Supplement: Supplementary Table 1 [file aging-17-206221-s002.pdf]

Supplementary Table

Supplementary Table 1. Epigenetic Biomarker Proxy (EBP) Analysis Between Baseline (0 months) and 12 Months Following Supplementation. The first column shows the epigenetic biomarker proxy assessed. Columns 2 and 3 report the marginal mean values for each time point. These values were adjusted for confounding factors technical variability such as age, and array type. The p-value column depicts the p-value of the Wilcoxon-rank sum test performed between baseline and 12 months. The final column depicts the adjusted p-value by using the false discovery rate (FDR) adjustment. The table includes 396 EBPs.

	Mean		0 vs 12 months	
	**0 months**	**12 months**	**p-value**	**FDR**
**deoxycholic acid glucuronide**	0.09521349614	0.01926854981	0.04136160471	1
**TTHY_HUMAN**	-0.01787292975	0.002262820717	0.04136160471	1
**beta-cryptoxanthin**	-0.02152434138	0.0230126822	0.07510688278	1
**ergothioneine**	-0.0130903173	-0.004331751329	0.08447481424	1
**4-acetamidophenol**	0.06218145268	0.02059019667	0.08447481424	1
**IF1AX_HUMAN_IF1AY_HUMAN**	0.1187914633	0.07511167789	0.1247717199	1
**dodecenedioate**	-0.01671622674	-0.003735426377	0.1422928156	1
**cysteine s-sulfate**	0.004629092434	-0.005284178479	0.149800547	1
**MYOC_HUMAN**	-0.02538097517	-0.003411835789	0.1741093598	1
**IBP5_HUMAN**	-0.01111721085	0.003172535683	0.1918552587	1
**cis-3,4-methyleneheptanoylcarnitine**	-0.03117731868	0.002802896031	0.2059999892	1
**PLMN_HUMAN**	-0.01285262518	-0.00272200703	0.2108768051	1
**trans-3,4-methyleneheptanoate**	-0.01572439032	-0.001259150751	0.2208761433	1
**LYSC_HUMAN**	-0.01071029253	-0.005924461983	0.2259995395	1
**PON1_HUMAN**	-0.02415664192	0.006106702842	0.2259995395	1
**arachidoylcarnitine**	0.03208989317	0.008411536511	0.231205966	1
**GRN_HUMAN**	-0.007761660016	-0.001692921048	0.2364958031	1
**salicylate**	-0.0318091145	-0.01288575272	0.2418694073	1
**cystine**	0.01731686939	-0.0000964707368	0.2528692218	1
**10-undecenoate**	-0.01387633951	-0.004503585043	0.264207768	1
**3-hydroxydodecanedioate***	-0.007490774196	-0.0002052763238	0.264207768	1
**H4_HUMAN**	-0.01529916186	-0.001277547441	0.2758869914	1
**ribitol**	0.003925455011	0.001988467737	0.2818548481	1
**THRB_HUMAN**	-0.004293647915	0.01420904353	0.2818548481	1
**glycosyl-N-behenoyl-sphingadienine**	-0.007524278202	0.003520880956	0.2940477923	1
**eicosenedioate**	-0.01847332257	-0.005341254794	0.2940477923	1
**F10A1_HUMAN**	0.03744465736	0.01937536847	0.2940477923	1
**ITIH3_HUMAN**	-0.02623582386	-0.01072724112	0.2940477923	1

**2-methylcitrate/homocitrate**	-0.02813440407	-0.0115981768	0.3002730913	1
**indolelactate**	0.05467739248	0.03169813047	0.3065843675	1
**gamma-glutamyltyrosine**	-0.007694903346	-0.003614215427	0.3065843675	1
**BMP1_HUMAN**	-0.006411807805	0.002223433066	0.3065843675	1
**2-oxindole-3-acetate**	-0.01721888668	-0.01043444506	0.3129816539	1
**PON1_HUMAN**	-0.01280620599	-0.004620669967	0.3194649531	1
**1-palmitoyl-2-docosahexaenoyl-GPE**	-0.01992933466	-0.007940208012	0.3260342379	1
**1-margaroyl-GPE**	-0.01835847447	-0.0042041271	0.33268945	1
**GPX3_HUMAN**	-0.02186469303	0.007206006385	0.33268945	1
**taurocholenate sulfate***	-0.02777293117	-0.006527503121	0.3394305005	1
**sphingomyelin**	-0.03574589937	-0.01884072551	0.3394305005	1
**IBP2_HUMAN**	-0.01913602535	-0.01118384801	0.3394305005	1
**LYSC_HUMAN**	-0.01128067127	-0.002719429248	0.3394305005	1
**PGK1_HUMAN**	0.02405111675	0.002856418465	0.3394305005	1
**1-palmitoyl-2-eicosapentaenoyl-GPE**	-0.01626459169	0.00573745307	0.3462572689	1
**APOF_HUMAN**	-0.02655432662	-0.00004982877665	0.353169603	1
**sphingomyelin**	-0.03815752943	-0.02805092348	0.3601673188	1
**PLMN_HUMAN**	-0.003705730379	0.0009144782042	0.3601673188	1
**sebacate**	-0.0229189925	-0.001349728651	0.3672502	1
**N-delta-acetylornithine**	-0.02014657162	-0.0224597498	0.3672502	1
**phenylalanine**	-0.002559750217	0.0004029856423	0.3672502	1
**CSPG2_HUMAN**	-0.03220528088	0.0009592195763	0.3744179979	1
**3beta-hydroxy-5-cholenoate**	0.01021087053	0.008582375557	0.3816704308	1
**PA2GA_HUMAN**	-0.0460750291	-0.002356349687	0.3890071844	1
**ALBU_HUMAN**	0.01245659717	0.003448553838	0.3890071844	1
**FETUA_HUMAN**	-0.002544661916	0.0006495502442	0.3890071844	1
**ERF1_HUMAN**	0.02144751519	0.02131754026	0.3964279109	1
**CBPB2_HUMAN**	-0.01936306127	-0.008518487733	0.3964279109	1
**N-acetylglucosamine/N-acetylgalactosamine**	-0.01706500515	-0.01049056794	0.4039322295	1
**carnitine**	-0.01122231587	-0.002699078344	0.4115197256	1
**2-aminoheptanoate**	-0.01217482591	0.004210227323	0.4115197256	1
**carotene diol**	-0.0005344990404	0.01392051032	0.4115197256	1
**BMP1_HUMAN**	0.0084700422	-0.001926858747	0.4115197256	1
**phenylacetylglycine**	0.009827523587	0.00894449021	0.4191899511	1
**5alpha-pregnan-3beta,20beta-diol monosulfate**	-0.03808263306	-0.0005983687951	0.4191899511	1

**FETUA_HUMAN**	-0.00114524397	0.0002363069786	0.4191899511	1
**HGFA_HUMAN**	-0.009020385942	-0.009849948578	0.4191899511	1
**ITIH3_HUMAN**	-0.001335996789	0.001722852853	0.4191899511	1
**APOF_HUMAN**	-0.02921141817	-0.01614905947	0.4191899511	1
**CSPG2_HUMAN**	-0.02518437442	-0.004033361175	0.4269424242	1
**PON1_HUMAN**	-0.001240246558	0.01396261538	0.4269424242	1
**LYSC_HUMAN**	-0.01248833925	-0.01008061005	0.4347766292	1
**beta-citrylglutamate**	-0.001105186113	-0.003364409352	0.4426920165	1
**p-cresol glucuronide***	0.02011590208	-0.01430107298	0.4426920165	1
**IBP2_HUMAN**	-0.02289854482	-0.007659849809	0.4426920165	1
**RNAS4_HUMAN**	-0.003559472038	-0.002767522607	0.4426920165	1
**4-guanidinobutanoate**	0.04703870818	0.04203380159	0.4506880026	1
**uridine**	-0.0211908296	0.005462500276	0.4587639698	1
**dihydroorotate**	0.007926341108	-0.0008497823571	0.4587639698	1
**Total_cholesterol**	-4.624280001	-3.83459081	0.4587639698	1
**PGK1_HUMAN**	0.006871433023	-0.00120142666	0.4587639698	1
**CFAD_HUMAN**	-0.005989975873	0.009261831939	0.4587639698	1
**PON1_HUMAN**	-0.01883224515	-0.008347055662	0.4669192668	1
**glycochenodeoxycholate 3-sulfate**	0.006168702684	0.003979502599	0.4669192668	1
**1-palmitoyl-2-arachidonoyl-GPE**	-0.01896009868	0.001702559206	0.4751532079	1
**docosahexaenoylcarnitine**	-0.006825679693	-0.001072570645	0.4751532079	1
**PON1_HUMAN**	-0.02925679353	-0.02201158276	0.4751532079	1
**H2B1_HUMAN**	-0.01374356896	-0.00489090163	0.4751532079	1
**ITIH3_HUMAN**	-0.01703477983	-0.007921790686	0.4751532079	1
**FBLN3_HUMAN**	-0.009602824991	0.006279682175	0.4751532079	1
**MXRA5_HUMAN**	0.003772095216	0.01861752144	0.4834650739	1
**RNAS1_HUMAN**	-0.0120334541	-0.002327662933	0.4834650739	1
**CBPB2_HUMAN**	-0.003975206171	-0.01876349819	0.4918541113	1
**CMPF**	-0.0459991495	0.0003921826782	0.4918541113	1
**N-acetylmethionine**	-0.007564390664	0.001117553943	0.4918541113	1
**CCL14_HUMAN**	-0.01614636665	-0.004417319577	0.4918541113	1
**PON1_HUMAN**	-0.008115168108	-0.006001703325	0.4918541113	1
**vanillylmandelate**	-0.02712158899	-0.008818375362	0.5003195333	1
**F10A1_HUMAN**	0.05436068896	0.03974745911	0.5088605191	1
**myristoylcarnitine**	-0.01267422048	-0.01062516238	0.5088605191	1
**1-linoleoyl-2-arachidonoyl-GPC**	0.01325855824	0.006246492012	0.5088605191	1
**3-4-hydroxyphenyl-lactate**	0.01799806996	0.007420691025	0.5088605191	1

**CRDL1-4_HUMAN**	-0.01187393316	-0.003257398816	0.5088605191	1
**SLPI_HUMAN**	0.01049731583	0.003413850279	0.5088605191	1
**HAP28_HUMAN**	0.004741626397	-0.001301230226	0.5088605191	1
**N-acetylalliin**	-0.01239847646	0.001629284894	0.5174762146	1
**N-carbamoylalanine**	0.03998421759	0.03674482344	0.5174762146	1
**carnitine of C10H14O2**	-0.003694305313	0.001478593931	0.5174762146	1
**sphingomyelin**	-0.09544509045	-0.1153190864	0.5261657322	1
**1-1-enyl-palmitoyl-2-linoleoyl-GPC**	-0.00136050132	-0.0002234433661	0.5261657322	1
**NOTUM_HUMAN**	-0.02566314436	0.001418268075	0.5349281514	1
**iminodiacetate**	0.002740393609	0.00488869225	0.5349281514	1
**tyramine O-sulfate**	0.005849932331	-0.003944932793	0.5349281514	1
**4-hydroxyphenylacetylglutamine**	-0.0372157041	-0.004803592133	0.5349281514	1
**3-hydroxyoleoylcarnitine**	-0.01313964753	-0.002863556827	0.5349281514	1
**methionine sulfone**	0.01217258693	0.01350053248	0.5437625185	1
**PCOC1_HUMAN**	-0.001422925623	0.001689702161	0.5437625185	1
**CO6A3_HUMAN**	0.02037644764	0.0001615236839	0.5437625185	1
**3-methylcytidine**	-0.00172083939	0.005256916525	0.5526678473	1
**tryptophan betaine**	-0.01798027853	-0.006821874704	0.5526678473	1
**behenoyl dihydrosphingomyelin**	-0.01179689509	-0.002219379243	0.5526678473	1
**citrulline**	0.0105840359	-0.0003094816966	0.5526678473	1
**gentisate**	0.003684641297	0.002568870161	0.5616431191	1
**21-hydroxypregnenolone disulfate**	-0.003942363423	0.02211962529	0.5616431191	1
**PON1_HUMAN**	0.003102532734	0.007510234529	0.5616431191	1
**COIA1_HUMAN**	-0.001246887732	0.001142410826	0.5616431191	1
**quinate**	-0.02108596122	0.01053968786	0.5706872829	1
**5-hydroxyhexanoate**	-0.01307891992	-0.003204525244	0.5706872829	1
**CCL16_HUMAN**	-0.009484502955	-0.00556382667	0.5706872829	1
**2-hydroxy-4-methylthio-butanoic acid**	0.01285385702	0.004055667327	0.5797992561	1
**branched-chain**	-0.001676735102	-0.0000485145252 4	0.5797992561	1
**PCOC1_HUMAN**	-0.002400245553	-0.005958733023	0.5797992561	1
**PON1_HUMAN**	-0.00115005082	-0.01126286857	0.5889779245	1
**Smoking_PackYears**	-0.3773867767	-0.5421617508	0.5889779245	1
**PCOC1_HUMAN**	-0.001512936817	-0.003801401276	0.5889779245	1
**BUN**	-0.3489588783	0.4993756357	0.5982221427	1
**phenylacetylcarnitine**	-0.02282197684	-0.005116232616	0.5982221427	1
**glucuronide of piperine metabolite**	0.02558827999	0.03271228408	0.5982221427	1
**serotonin**	-0.004393564183	-0.0107027819	0.5982221427	1

**IBP6_HUMAN**	-0.01292064006	-0.005003766601	0.5982221427	1
**N-methylpipecolate**	0.01213120686	-0.01095523588	0.6075307345	1
**malonate**	0.004218631529	-0.001123583677	0.6075307345	1
**sucrose**	-0.01088769964	-0.01954424283	0.6075307345	1
**Lipid_HDL**	-0.2113849259	-0.8346921048	0.6075307345	1
**RNAS1_HUMAN**	-0.02128459742	-0.003250287967	0.6075307345	1
**1-pentadecanoyl-GPC**	0.01123602682	0.005298430012	0.6169024936	1
**hypoxanthine**	-0.00397493862	0.003530041456	0.6169024936	1
**HbA1c**	-0.001094187447	-0.05222220277	0.6169024936	1
**PCOC1_HUMAN**	-0.009117782514	-0.009536472138	0.6169024936	1
**7-hydroxyindole sulfate**	-0.006989631676	0.001260323411	0.6263361834	1
**carotene diol**	-0.00248095836	0.01327366633	0.6263361834	1
**glycine conjugate of C10H14O2**	-0.007344401229	0.006480241886	0.6263361834	1
**androsterone glucuronide**	0.01254415169	0.007872787978	0.6358305382	1
**lyxonate**	-0.02417287576	-0.001971750824	0.6358305382	1
**glutamine conjugate of C7H12O2***	-0.02100671585	0.003154501771	0.6358305382	1
**retinol_vitamin A**	0.01235473448	-0.008253217224	0.6358305382	1
**glutamate**	0.03898497633	0.0297465838	0.6358305382	1
**SLPI_HUMAN**	0.001805903641	0.003051814161	0.6358305382	1
**1-palmitoyl-GPC**	-0.004301871921	0.004407061717	0.6453842629	1
**N-oleoyltaurine**	0.009295520558	-0.005011623514	0.6453842629	1
**carotene diol**	-0.01064540802	-0.003231234442	0.6453842629	1
**hydroxyasparagine****	-0.04546731167	-0.01681801525	0.6453842629	1
**BMP1_HUMAN**	-0.0009616211434	0.001101835292	0.6453842629	1
**2-hydroxyglutarate**	0.003779134298	-0.0002734598228	0.6549960342	1
**4-hydroxyglutamate**	-0.004235470846	0.009525204186	0.6646645006	1
**behenoyl sphingomyelin**	-0.007128648829	-0.0118510328	0.6646645006	1
**pantothenate**	0.009301085151	0.003499720888	0.6646645006	1
**BMP1_HUMAN**	0.00527825787	-0.001151059982	0.6646645006	1
**PCOC1_HUMAN**	-0.001824963364	-0.01341448223	0.6646645006	1
**BMP1_HUMAN_TLL1_HUMAN**	0.008279509358	0.009116943316	0.6646645006	1
**21-hydroxypregnenolone monosulfate**	0.02047844821	0.02505541364	0.6743882834	1
**3-methoxytyramine sulfate**	-0.0004267236607	0.001683939357	0.6743882834	1
**N,N,N-trimethyl-5-aminovalerate**	0.001942971889	0.0003579691883	0.6743882834	1
**3,5-dichloro-2,6-dihydroxybenzoic acid**	0.00514940974	-0.0006568930813	0.6743882834	1
**spermidine**	-0.001055948049	0.0004256326116	0.6743882834	1

**adenosine**	0.005796652126	-0.000718190967	0.6743882834	1
**PON1_HUMAN**	0.001434378314	0.002342310376	0.6743882834	1
**salicyluric glucuronide***	-0.02802135372	-0.04918217544	0.6841659768	1
**5-methyluridine_ribothymidine**	-0.004379219515	-0.005729769515	0.6841659768	1
**N,N-dimethyl-5-aminovalerate**	-0.01225383669	-0.007222532822	0.6841659768	1
**indole-3-carboxylate**	-0.003843265705	0.004234424085	0.6939961488	1
**1,2-dilinoleoyl-GPC**	0.004504610847	0.006583628895	0.6939961488	1
**hexanoylcarnitine**	-0.02796705242	-0.005658845869	0.7038773417	1
**1-stearoyl-2-adrenoyl-GPC**	0.01036727773	0.004905264399	0.7038773417	1
**1,2-dipalmitoyl-GPC**	-0.000297902569	-0.002856682566	0.7138080727	1
**N2-acetyllysine**	0.004992108324	0.0210842406	0.7138080727	1
**N-linoleoyltaurine***	0.003934595201	0.009501637368	0.7138080727	1
**2,4-di-tert-butylphenol**	-0.01311637984	-0.00690386642	0.7138080727	1
**urate**	0.04598383487	0.03331258591	0.7138080727	1
**Lipid_LDL**	-1.651187072	-1.146644672	0.7138080727	1
**androsterone sulfate**	0.02676018456	-0.006322034763	0.7237868346	1
**N-acetylglutamine**	-0.005589500902	-0.001707110232	0.7237868346	1
**3-ureidopropionate**	0.01204562577	-0.0007545460689	0.7237868346	1
**beta-hydroxyisovalerate**	0.02787143188	0.01442080084	0.7237868346	1
**leucine**	0.008253622193	0.01104221869	0.7237868346	1
**H4_HUMAN**	-0.0113587229	0.009100303614	0.7237868346	1
**4-methoxyphenol sulfate**	-0.02478983565	-0.01084022182	0.7338120965	1
**guanidinosuccinate**	0.01181040988	-0.0008179752311	0.7338120965	1
**N-acetyl-3-methylhistidine***	0.03452016005	0.004126826187	0.7338120965	1
**hydroxybutyrylcarnitine**	-0.009805178995	-0.001158050556	0.7338120965	1
**malate**	0.0002244056781	-0.002880515035	0.7338120965	1
**urea**	0.003363306669	-0.0002111459859	0.7338120965	1
**CIRBP_HUMAN**	0.005170159797	-0.01428493104	0.7338120965	1
**1,5-anhydroglucitol**	-0.002411641815	-0.001954732556	0.7438823044	1
**phenylacetylglutamine**	-0.000811366007	0.002898187241	0.7438823044	1
**androsterone glucuronide**	0.03994957598	-0.005667850182	0.7438823044	1
**argininate***	0.01454677278	0.01095808085	0.7438823044	1
**succinimide**	0.009684818479	0.02629895971	0.7438823044	1
**2-hydroxyphytanate***	-0.001163002157	-0.0003021092455	0.7438823044	1
**trans-2-hexenoylglycine**	-0.002921676589	-0.003093324553	0.7438823044	1
**N-acetyl-2-aminooctanoate***	0.01768782038	0.01202556645	0.7438823044	1
**creatinine**	0.04581507263	0.03460854335	0.7438823044	1

**ornithine**	-0.002709040851	0.0003870842184	0.7438823044	1
**IBP6_HUMAN**	-0.001736859328	0.00000340195048	0.7438823044	1
**11-ketoetiocholanolone glucuronide**	-0.003488207661	-0.002263474378	0.7539958819	1
**1-stearoyl-2-dihomo-linolenoyl-GPC**	-0.01080973943	0.0004904677984	0.7539958819	1
**catechol glucuronide**	-0.006638382658	-0.001979816122	0.7539958819	1
**levulinoylcarnitine**	-0.03232038881	-0.02298504915	0.7539958819	1
**Red_Cell_Dist_Width**	-0.02768352386	0.04248710144	0.764151231	1
**N-acetyl-cadaverine**	0.01112135523	-0.0004995026283	0.764151231	1
**N-stearoyl-sphingosine**	0.008081239299	-0.005976290188	0.764151231	1
**FVC**	0.03152089063	0.1199313496	0.764151231	1
**Total_Bilirubin**	0.01127231823	0.02543383211	0.764151231	1
**H4_HUMAN**	-0.001871166025	0.007065705233	0.764151231	1
**IBP6_HUMAN**	0.00567077806	-0.004434789513	0.764151231	1
**CBPB2_HUMAN**	-0.00522181652	-0.0034306589	0.764151231	1
**gamma-glutamylglycine**	0.005280937564	-0.005127218066	0.7743467329	1
**glutamine_degradant***	0.03157239674	0.04505798109	0.7743467329	1
**chiro-inositol**	0.002812665617	-0.001241232021	0.7743467329	1
**cinnamoylglycine**	-0.01842136169	0.006415178086	0.7743467329	1
**1-methyl-5-imidazoleacetate**	-0.002779697985	-0.0007840293948	0.7743467329	1
**1-margaroyl-2-arachidonoyl-GPC**	0.006375764623	-0.00434094222	0.7743467329	1
**S-carboxyethylcysteine**	-0.004932428975	-0.003458015037	0.7743467329	1
**N1-methyladenosine**	-0.006147127738	0.003777355238	0.7743467329	1
**alpha-ketoglutarate**	-0.004347028003	0.001011221059	0.7743467329	1
**Creatinine**	0.01790761186	0.05981818206	0.7845807485	1
**N-acetylglutamate**	0.002605341386	0.003398197013	0.7845807485	1
**dimethyl sulfone**	0.007322108301	-0.008238885098	0.7845807485	1
**thyroxine**	-0.01092529013	-0.0030217041	0.7845807485	1
**riboflavin_vitamin B2**	0.002714678224	0.0003114881634	0.7845807485	1
**mannose**	0.00004522457734	-0.008788299515	0.7845807485	1
**IBP2_HUMAN**	-0.000774833693	-0.01731543688	0.7845807485	1
**dehydroepiandrosterone sulfate**	0.007183633549	-0.01714418736	0.7948516193	1
**galactonate**	0.008612631455	-0.003267978425	0.7948516193	1
**3-methyladipate**	-0.02447109347	-0.02068788117	0.7948516193	1
**6-oxopiperidine-2-carboxylate**	0.0005515196986	-0.000091081602	0.7948516193	1
**1-pentadecanoyl-2-linoleoyl-GPC**	0.001947939006	0.0008825952121	0.7948516193	1
**undecenoylcarnitine**	0.000201498693	0.003840921933	0.7948516193	1
**acetoacetate**	-0.01205742786	-0.0004589688844	0.7948516193	1

**PCOC1_HUMAN**	-0.01174933553	-0.001244751101	0.7948516193	1
**CBPB2_HUMAN**	-0.004494451143	-0.01641263144	0.8051576685	1
**2-hydroxysebacate**	-0.009769557297	-0.005490642275	0.8051576685	1
**3-hydroxyoctanoylcarnitine**	-0.005041560113	0.006622790813	0.8051576685	1
**alpha-tocopherol**	-0.004557641039	0.002599479501	0.8051576685	1
**CCL18_HUMAN**	-0.003001495916	0.0001111969152	0.8051576685	1
**sphinganine-1-phosphate**	-0.009261750683	-0.01428088572	0.8154972013	1
**2-hydroxybutyrate/2-hydroxyisobutyrate**	0.0009706868634	0.004240879096	0.8154972013	1
**1-myristoyl-2-arachidonoyl-GPC**	-0.006195463345	-0.01043604299	0.8154972013	1
**guanidinoacetate**	0.01012044055	0.01336450462	0.8154972013	1
**MMP19_HUMAN**	-0.02653603468	0.001681108828	0.8154972013	1
**MGP_HUMAN**	-0.001503776746	-0.005054307908	0.8154972013	1
**homoarginine**	0.006237072837	0.003347174475	0.8258685061	1
**1-docosahexaenoylglycerol**	-0.007467364725	0.004369252343	0.8258685061	1
**omeprazole**	0.008992665199	0.001327141259	0.8258685061	1
**ferulic acid 4-sulfate**	-0.003579196029	-0.004116613562	0.8258685061	1
**lithocholic acid sulfate**	-0.001507843427	-0.02491803623	0.8258685061	1
**menthol glucuronide**	0.002054607562	0.00626310139	0.8258685061	1
**FEV1**	0.01263564479	-0.0003738401406	0.8258685061	1
**androstenediol disulfate**	0.0254844542	0.02143930472	0.8362698549	1
**ximenoylcarnitine**	0.0104466661	0.001750193861	0.8362698549	1
**glucuronide of C12H22O4**	0.004362244735	-0.003100376177	0.8362698549	1
**glucuronide of C10H18O2**	0.01690139319	-0.01185645254	0.8362698549	1
**dihydroferulic acid sulfate**	0.00546823586	-0.007871151349	0.8362698549	1
**cystathionine**	-0.001756235618	-0.0119903382	0.8362698549	1
**gluconate**	0.01308601975	0.004552692306	0.8362698549	1
**IBP2_HUMAN**	-0.01788424862	0.0006890374773	0.8362698549	1
**IBP2_HUMAN**	-0.01219100786	-0.01232208865	0.8362698549	1
**AMBP_HUMAN**	-0.000221366484	-0.002371933696	0.8362698549	1
**3-methylxanthine**	0.01780127325	0.01388676794	0.8466995048	1
**lactosyl-N-palmitoyl-sphingosine**	0.0009454570552	0.01005823526	0.8466995048	1
**isocitric lactone**	-0.0006431301146	-0.01121163584	0.8466995048	1
**picolinate**	0.008181046817	-0.005672628929	0.8466995048	1
**estrone 3-sulfate**	0.01789460219	-0.004930396075	0.8466995048	1
**methylsuccinate**	0.009148350372	0.008826990704	0.8466995048	1
**lactose**	-0.0180915841	-0.01717273864	0.8466995048	1
**Direct_Bilirubin**	0.00346949145	-0.002952313422	0.8466995048	1

**CYTC_HUMAN**	0.004358411739	0.006052196772	0.8466995048	1
**PCOC1_HUMAN**	-0.001657621409	-0.003863431324	0.8466995048	1
**FETUA_HUMAN**	-0.00166548027	-0.002959093398	0.8466995048	1
**indoleacetylglutamine**	-0.01210635073	-0.01258944035	0.8571556983	1
**suberoylcarnitine**	-0.006500858173	-0.003736511793	0.8571556983	1
**3-hydroxyphenylacetoylglutamine**	0.004760548423	-0.0007677129168	0.8571556983	1
**3-hydroxyhippurate sulfate**	-0.008923533731	-0.004445531048	0.8571556983	1
**picolinoylglycine**	-0.01636589977	-0.008805926599	0.8571556983	1
**3-methoxytyrosine**	0.001078768735	-0.00101036535	0.8571556983	1
**uracil**	-0.0006571386716	0.008711979628	0.8571556983	1
**caffeine**	-0.004290710557	0.005980318959	0.8571556983	1
**BMP1_HUMAN**	0.001998726076	0.007976298157	0.8571556983	1
**CRP**	-0.5716600154	-0.8795429204	0.8676366644	1
**Triglyceride**	6.929036211	8.419515568	0.8676366644	1
**1-methylguanidine**	-0.001874769613	0.009839243521	0.8676366644	1
**2-methoxyhydroquinone sulfate**	0.002404068687	-0.005701643001	0.8676366644	1
**xanthine**	-0.0007086834362	0.004437497101	0.8676366644	1
**BMI**	-0.398626512	-0.4265069556	0.8781406193	1
**Mean_Corpus_Vol**	-0.005140046971	-0.07462663989	0.8781406193	1
**gamma-glutamylphenylalanine**	-0.006296186425	-0.008285952429	0.8781406193	1
**N-acetyl-1-methylhistidine***	0.01217369022	0.001264037307	0.8781406193	1
**IBP2_HUMAN**	0.008567214547	-0.01127486066	0.8781406193	1
**Hematocrit**	-0.02747061677	0.0117766837	0.8886657678	1
**Hemoglobin**	0.07655711043	0.02614875731	0.8886657678	1
**lanthionine**	0.0102448451	-0.01082469032	0.8886657678	1
**3-hydroxyadipate**	-0.002938183935	-0.00271150523	0.8886657678	1
**histidine**	-0.004385928722	-0.005372601937	0.8886657678	1
**PON1_HUMAN**	0.002665147356	-0.005966065352	0.8886657678	1
**vanillactate**	-0.01035991041	0.01043643217	0.8992103035	1
**citramalate**	-0.002211968243	-0.002238572268	0.8992103035	1
**1-palmitoyl-2-linoleoyl-GPC**	-0.00007248055106	0.0006533241959	0.8992103035	1
**choline phosphate**	0.005983431112	0.0006186251196	0.8992103035	1
**Glucose**	0.5460330662	-0.3422180118	0.9097724102	1
**threonate**	0.00324543569	0.004827587657	0.9097724102	1
**indolebutyrate**	-0.006053224109	-0.008844893767	0.9097724102	1
**mannonate***	-0.01262400715	-0.02350412979	0.9097724102	1
**dopamine 4-sulfate**	0.01279533244	-0.009267382023	0.9097724102	1

**methyl vanillate sulfate**	-0.0004848661104	0.0002661157931	0.9097724102	1
**PCOC1_HUMAN**	-0.00341107377	-0.000744108349	0.9097724102	1
**IBP2_HUMAN**	0.005669370068	0.001659763185	0.9097724102	1
**IBP2_HUMAN**	0.006907153976	-0.008835295104	0.9203502627	1
**acetylcarnitine**	0.009398454927	-0.01214770982	0.9203502627	1
**furaneol sulfate**	0.0000694288123 6	0.0007145409782	0.9203502627	1
**decadienedioic acid**	-0.001261484486	-0.00507642598	0.9203502627	1
**ibuprofen**	-0.0001368705989	-0.0006268682788	0.9203502627	1
**Liver_ALB**	0.002274890215	0.01445233155	0.9309420277	1
**IGF1_HUMAN**	0.005102323727	-0.006883971718	0.9309420277	1
**indoleacetate**	0.006320481392	0.003035460761	0.9309420277	1
**nicotinamide riboside**	-0.0001972355007	-0.0006034880904	0.9309420277	1
**deoxycarnitine**	0.002142700224	0.01738635548	0.9309420277	1
**dimethylarginine**	-0.005413716245	-0.007170778027	0.9309420277	1
**arabitol/xylitol**	-0.02949414595	-0.01379063825	0.9309420277	1
**6-bromotryptophan**	0.01093570922	0.003640162619	0.9309420277	1
**5-hydroxymethyl-2-furoylcarnitine***	-0.00876184371	-0.02709432722	0.9309420277	1
**TLL1_HUMAN**	0.009535319377	0.006381144214	0.9309420277	1
**hippurate**	-0.005484805353	-0.01370285513	0.9415458647	1
**octadecanedioylcarnitine**	-0.001920106126	0.01539395498	0.9415458647	1
**3-hydroxyindolin-2-one sulfate**	-0.005583347423	0.002408696987	0.9415458647	1
**cortisol**	-0.00276673417	-0.008359807866	0.9415458647	1
**tryptophan**	0.0023194349	0.004385611456	0.9415458647	1
**Liver_ALP**	-0.5202097593	-0.3278945479	0.9415458647	1
**PCOC1_HUMAN**	-0.005815337439	-0.005311710092	0.9415458647	1
**isobutyrylcarnitine**	0.003634404402	0.007856259835	0.9521599269	1
**N-acetyltyrosine**	0.001499157086	-0.001431785845	0.9521599269	1
**vanillic acid glycine**	0.003358128912	0.006815753867	0.9521599269	1
**trans-4-hydroxyproline**	0.001050541509	0.0002013719495	0.9521599269	1
**cholesterol**	-0.005909076232	-0.01143557609	0.9521599269	1
**proline**	0.002193867386	0.006171374381	0.9521599269	1
**serine**	0.007419858508	0.006975964788	0.9521599269	1
**HGFA_HUMAN**	0.0008048788769	-0.0004928273272	0.9521599269	1
**tiglylcarnitine**	0.00890605837	-0.005878639955	0.9627823626	1
**5-galactosylhydroxy-lysine**	-0.003127925539	-0.003650265848	0.9627823626	1
**3-methoxycatechol sulfate**	0.01158288223	0.004178811825	0.9627823626	1
**N-acetyl-isoputreanine**	-0.00688828385	-0.01325600412	0.9627823626	1

**hypotaurine**	0.007654375028	-0.0009594021392	0.9627823626	1
**MIME_HUMAN**	-0.009890481422	0.00003499864771	0.9627823626	1
**2-acetamidophenol sulfate**	0.02872467365	0.001920423765	0.9734113153	1
**eicosenoylcarnitine**	0.01246072377	0.004295425321	0.9734113153	1
**nicotinamide**	0.003200842443	0.006106192205	0.9734113153	1
**BMP1_HUMAN**	0.00141373686	0.0007753092263	0.9734113153	1
**MXRA5_HUMAN**	0.004216531297	0.00518780205	0.9734113153	1
**IBP6_HUMAN**	-0.0119728877	0.002213881099	0.9734113153	1
**xanthosine**	0.01606745582	0.004446333901	0.9840449255	1
**propionylcarnitine**	0.01211956139	0.002399190106	0.9840449255	1
**N-acetylcitrulline**	0.01512303957	0.01039833419	0.9840449255	1
**cyclo_gly-pro**	0.001540823751	-0.007246737006	0.9840449255	1
**ethyl alpha-glucopyranoside**	-0.005334414086	0.01143897747	0.9840449255	1
**3-amino-2-piperidone**	-0.01480953767	-0.02453794322	0.9840449255	1
**dimethylguanidino valeric acid**	0.0030760457	-0.003571011108	0.9840449255	1
**arabinose**	0.0009670106063	0.001658935387	0.9840449255	1
**SLPI_HUMAN**	-0.001111681457	-0.004872878765	0.9840449255	1
**BMP1_HUMAN**	0.00740404507	0.01128670942	0.9840449255	1
**PCOC1_HUMAN**	0.003017403408	0.004182820073	0.9840449255	1
**pregnanediol-3-glucuronide**	0.009017756051	0.0004788910054	0.9946813312	1
**alpha-CMBHC glucuronide**	-0.01787111475	-0.008056942645	0.9946813312	1
**stearoyl-arachidonoyl-glycerol**	0.0007911031877	0.001003212236	0.9946813312	1
**cytosine**	0.006018701057	0.004673232414	0.9946813312	1
**IBP2_HUMAN**	-0.0001295536412	-0.00359491755	0.9946813312	1
**CRDL1-4_HUMAN**	0.005410291199	0.008543464954	0.9946813312	1
**succinylcarnitine**	-0.003704064056	0.001034480478	0.9946813312	1
**linoleoylcarnitine**	0.01657949941	-0.001438207362	0.9946813312	1
**N-acetyl-S-allyl-cysteine**	-0.005829258957	-0.0007050492053	0.9946813312	1
**phenylacetylglutamate**	-0.001955216825	-0.004953423538	0.9946813312	1
**2,3-dihydroxy-2-methylbutyrate**	-0.01204520374	-0.01996415325	0.9946813312	1
**homovanillate**	-0.003007654632	-0.004217732784	0.9946813312	1
**CCL14_HUMAN**	-0.006253210793	-0.006710378206	0.9946813312	1
**PCOC1_HUMAN**	0.00571273161	0.001057456291	0.9946813312	1
**3-hydroxy-2-ethylpropionate**	-0.006379518365	-0.01231421488	1	1
**N1-methyl-2-pyridone-5-carboxamide**	-0.01157705713	-0.01546103826	1	1
**acetylspermidine**	0.01174885454	-0.0004996917207	1	1
**3-hydroxybutyroylglycine****	-0.01666987788	-0.002321167331	1	1

**glycine conjugate of C10H12O2***	0.001100153416	0.01073627757	1	1
