# Supplementary material for: Effects of a natural ingredients-based intervention targeting the hallmarks of aging on epigenetic clocks, physical function, and body composition: a single-arm clinical trial
Source: Aging (Albany NY). 2025 Mar 14;17(3):699–725. doi: 10.18632/aging.206221 (PMC11984428; doi:10.18632/aging.206221)
Supplement: Supplementary Table 2 [file aging-17-206221-s003.pdf]

SUPPLEMENTARY TABLE

Supplementary Table 2. Marioni protein biomarker analysis between baseline and 12 months.

	Mean		0 vs. 12 months	
	**0 months**	**12 months**	***P*-value**	**FDR**
**HGFA**	−0.004203092086	−0.00002423180198	0.006633638129	0.776135661
**CCL25.C.C**	0.002364087425	−0.0002437389103	0.04517312702	0.9989683987
**Smoking**	0.05098585389	−0.05589807382	0.08928440953	0.9989683987
**Complement.C5a**	−0.002406946082	−0.0005270524153	0.09389576275	0.9989683987
**CCL11**	0.001569555276	0.0000639118954	0.09707473754	0.9989683987
**CCL17**	0.002145944585	−0.002608575736	0.127691418	0.9989683987
**LFT**	−0.001969481142	−0.0005748644109	0.127691418	0.9989683987
**G.CSF**	0.00002570528647	−0.00196911118	0.1778664331	0.9989683987
**SLITRK5**	−0.002253284984	0.002162560076	0.1910861944	0.9989683987
**Complement.c9**	−0.003249777039	0.002421814867	0.1993575334	0.9989683987
**CD6**	0.000539745659	−0.002415556568	0.2078874653	0.9989683987
**Complement.C4**	0.0008952033478	0.002461451791	0.2166790143	0.9989683987
**FGF.21**	−0.002600517248	−0.002642970462	0.2226867873	0.9989683987
**L.selectin**	−0.001787397862	0.002164534726	0.2414228278	0.9989683987
**MMP.12**	0.002845722996	−0.0008490416694	0.2446503463	0.9989683987
**RARRES2**	0.0006662812642	−0.0008207870631	0.2893759682	0.9989683987
**FcRL2**	0.001409784616	−0.0003806847187	0.2967171324	0.9989683987
**Myeloperoxidase**	−0.001700098875	0.0009619427831	0.3079605512	0.9989683987
**NMNAT1**	0.0009248453747	−0.001046839759	0.3079605512	0.9989683987
**Alpha.L.iduronidase**	−0.001586732312	0.0005394910755	0.335277798	0.9989683987
**MMP.1.1**	0.002617914772	−0.0004795092825	0.3393039543	0.9989683987
**Waist.Hip.Ratio**	0.004785177115	0.002362838477	0.3474488829	0.9989683987
**Testican.2**	0.001418897395	0.0001742450372	0.3515676019	0.9989683987
**PIGR**	0.0008219686285	−0.0002180970136	0.3598973853	0.9989683987
**ESM.1**	−0.000576849828	0.0005601781908	0.4124403625	0.9989683987
**HDL.Cholesterol**	−0.01181922425	0.000002421792444	0.4170151527	0.9989683987
**B2.microglobulin**	0.003914250702	−0.0004911990806	0.4450856538	0.9989683987
**CCL21**	−0.0005963455388	−0.0004011925487	0.4595153953	0.9989683987
**FAP**	−0.0001376186235	0.0003160726244	0.4595153953	0.9989683987
**MIA**	0.00001389760461	−0.0009370849232	0.4643828978	0.9989683987
**Interleukin.19**	−0.00005526310484	−0.0004332867977	0.4742034356	0.9989683987
**Coagulation.factor.VII**	0.003018531584	0.003091150809	0.4791561718	0.9989683987
**Semaphorin.3E**	−0.0002407264728	0.00003832858881	0.4791561718	0.9989683987
**LGALS3BP**	0.00008624623521	0.0003827604661	0.4891457976	0.9989683987
**BMP.1**	−0.00240163659	−0.001623841147	0.5043381406	0.9989683987
**GHR**	−0.002040970852	−0.0006354712703	0.5043381406	0.9989683987
**CCL22**	0.0004679041346	−0.0003383886162	0.5197757608	0.9989683987
**NTRK3**	−0.002482866877	0.0001828863074	0.5197757608	0.9989683987
**TNFRSF17**	−0.0008848719987	−0.0001668868935	0.5197757608	0.9989683987
**CCL18**	−0.00118479946	0.001843641076	0.5302012868	0.9989683987
**FCER2**	0.0009996415821	−0.0004107485766	0.5302012868	0.9989683987
**Tryptase.beta.2**	−0.009750982018	−0.002955007247	0.5302012868	0.9989683987
**VEGFA**	0.001761861761	0.001340692446	0.5729382548	0.9989683987

**NRTK3**	0.0003012171188	0.0001495477023	0.5783931804	0.9989683987
**N.CDase**	0.002411467873	0.00935735488	0.5838724	0.9989683987
**Osteomodulin**	0.001871324262	−0.00017438412	0.5949027425	0.9989683987
**THBS2**	0.0004041928107	−0.002311829086	0.5949027425	0.9989683987
**BCAM**	0.000003496856842	−0.00001249792955	0.6060272831	0.9989683987
**IGFBP.1**	0.002664630607	0.003683271563	0.6342370447	0.9989683987
**SERPIN.A3**	0.003140451133	0.0008758347599	0.6342370447	0.9989683987
**MMP.2**	0.000008367060625	−0.0000792192152	0.6399451274	0.9989683987
**LY9**	0.002719537659	0.002161660627	0.6514251765	0.9989683987
**CXCL11.soma**	0.0007340883083	−0.0003227192082	0.6571965641	0.9989683987
**Resistin**	0.0002396798435	0.001550441076	0.6571965641	0.9989683987
**Body.Fat..**	−1.216026354	−0.2231436556	0.6688005903	0.9989683987
**SIGLEC1**	−0.0001177740104	−0.00180246272	0.6688005903	0.9989683987
**SKR3**	0.00001285003897	−0.002576840009	0.6746326279	0.9989683987
**ADAMTS**	0.003942952981	0.003928218319	0.6863552226	0.9989683987
**NEP**	−0.003103583437	0.0009963272374	0.7040807194	0.9989683987
**HCII**	0.001086743038	0.0008045524871	0.7159883899	0.9989683987
**OSM**	0.001572286871	−0.001098441165	0.7159883899	0.9989683987
**SMPD1**	0.002112516179	0.001853556137	0.7219684478	0.9989683987
**ENPP7**	0.002613330293	0.003970865634	0.7400095086	0.9989683987
**HGFI**	0.04255047106	0.04580961298	0.7400095086	0.9989683987
**CD163**	0.0009508661162	−0.001900454481	0.7642869187	0.9989683987
**CXCL9**	0.0004576005966	0.0008835725259	0.776514776	0.9989683987
**EZR**	0.00005323461148	−0.0003401201723	0.7826497483	0.9989683987
**MMP.9**	−0.000681185814	−0.0005637678863	0.7826497483	0.9989683987
**GPIba**	−0.0006501529099	−0.0009839887479	0.7887982685	0.9989683987
**ICAM5**	−0.003377737211	−0.001299728137	0.7887982685	0.9989683987
**PAPP.A**	−0.001186602545	−0.001643318436	0.7887982685	0.9989683987
**Adiponectin**	−0.000368786407	−0.003670844601	0.7949599725	0.9989683987
**E.selectin**	−0.0008755952635	−0.0007930053268	0.7949599725	0.9989683987
**GDF.8**	0.0002305888132	0.0002630791744	0.7949599725	0.9989683987
**MRC2**	−0.0003441140358	0.000241747697	0.8073214634	0.9989683987
**CRTAM**	−0.0003649384859	0.0008722371158	0.8135205102	0.9989683987
**Galectin.4**	−0.0002285249335	−0.0001310805028	0.8197312605	0.9989683987
**Lymphotoxin.abeta**	−0.001000085492	−0.004109682487	0.8259533386	0.9989683987
**CHIT.1**	0.0001064523224	0.001253765024	0.8321863666	0.9989683987
**Contactin.4**	0.00122158863	0.0008709213154	0.8321863666	0.9989683987
**NCAM.120**	0.001472576064	−0.0002932360989	0.8321863666	0.9989683987
**Aminoacylase.1**	0.001675816685	−0.001459171278	0.8446837506	0.9989683987
**Body.Mass.Index**	−0.01829311343	−0.0128794876	0.8572203494	0.9989683987
**MMP.1**	−0.0001887707847	−0.0003103901232	0.8572203494	0.9989683987
**Trypsin.2**	−0.001670578608	−0.002872303469	0.8572203494	0.9989683987
**CDL5**	−0.0005564806795	0.001898859675	0.8697930701	0.9989683987
**Granzyme.A**	−0.0009004787999	−0.00180643731	0.8697930701	0.9989683987
**Stanniocalcin.1**	−0.002183077473	0.00195805378	0.8697930701	0.9989683987
**TGF.alpha**	0.0002829634784	−0.001320731373	0.8697930701	0.9989683987
**Relative.IL6.Level**	0.0126283489	0.01777560992	0.8697930701	0.9989683987
**TPO**	0.000516942982	−0.0004800328284	0.8887130474	0.9989683987

**Ectodysplasin.A**	−0.0005005245063	−0.00007858233604	0.9013623677	0.9989683987
**Lysozyme.C**	−0.0002508442498	−0.00007384676591	0.9013623677	0.9989683987
**Alcohol**	−0.03447228293	−0.08453733175	0.9076966385	0.9989683987
**TNFRSF1B**	−0.0002531632742	−0.0002662013871	0.9140367846	0.9989683987
**SHBG**	0.001450253694	0.0001245994045	0.9203824056	0.9989683987
**CD48.antigen**	−0.0005786074031	−0.002001623108	0.9267331002	0.9989683987
**CLEC11A.e2**	−0.00523528939	−0.003384806367	0.9330884662	0.9989683987
**FCGR3A**	−0.0006931375727	−0.00293786917	0.9330884662	0.9989683987
**Granulysin**	0.0003857477398	0.00001546427019	0.9394481002	0.9989683987
**GZMA**	−0.0006083701915	−0.00002283059874	0.9458115984	0.9989683987
**WFIKKN2**	−0.000192622704	0.001309446329	0.9458115984	0.9989683987
**Epigenetic.Age..Zhang.**	0.06837951592	0.1544228142	0.952178556	0.9989683987
**CXCL10.soma**	0.000530649964	−0.00005319367948	0.952178556	0.9989683987
**CXCL11**	−0.001922826117	0.003847603819	0.952178556	0.9989683987
**S100.A9**	−0.001809123652	−0.0009185710265	0.952178556	0.9989683987
**CXCL10**	−0.002239725134	−0.002218975739	0.9585485674	0.9989683987
**NOTCH1**	−0.0001156664781	0.0003349860589	0.9585485674	0.9989683987
**VCAM1**	−0.0003345048158	0.00003166906158	0.9585485674	0.9989683987
**Afamin**	−0.000354242315	−0.0002201612204	0.9649212268	0.9989683987
**HGF**	0.0001379774814	0.0002887524682	0.9712961276	0.9989683987
**CRP**	−0.001499514853	−0.000571591246	0.9776728628	0.9989683987
**EN.RAGE**	−0.001079737208	0.0006186750341	0.9776728628	0.9989683987
**CLEC11A.e1**	−0.003310912633	−0.002682490204	0.9840510252	0.9989683987
**IGFBP.4**	−0.0001903608078	0.0004681879562	0.9840510252	0.9989683987
**CD209.antigen**	0.00112310137	−0.002100367834	0.9904302072	0.9989683987
**Insulin.receptor**	−0.0009746132488	−0.0005752805747	1	1

The first column details all Marioni proteins assessed between baseline and 12 months. The following columns (columns 2 and 3) report the predicted marginal mean values at each of the time points. The *p*-value column depicts the *p*-value of the Wilcoxon-rank sum test performed between baseline and 12 months. The final column depicts the adjusted *p*-value by using the false discovery rate (FDR) adjustment.
